# *QuickStats:* Age-Adjusted Prevalence of Total, Diagnosed, and Undiagnosed Diabetes* Among Adults Aged ≥20 Years — National Health and Nutrition Examination Survey, 1999–2000 to 2015–2016^†^

**DOI:** 10.15585/mmwr.mm6739a9

**Published:** 2018-10-05

**Figure:**
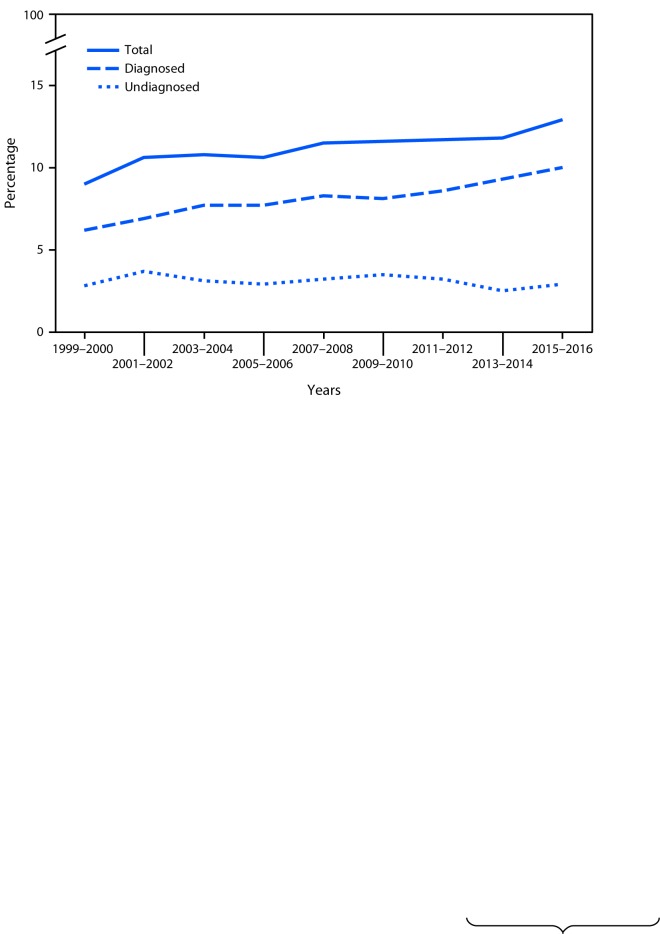
From 1999–2000 to 2015–2016, the prevalence of total diabetes increased from 9.0% to 12.9%. The prevalence of diagnosed diabetes increased from 6.2% to 10.0%. The prevalence of undiagnosed diabetes was 2.8% in 1999–2000 and 2.9% in 2015–2016 with no significant change over this period.

**Source:** CDC/NCHS National Health and Nutrition Examination Survey. https://www.cdc.gov/nchs/nhanes/index.htm.

